# A systematic review of non-productivity-related animal-based indicators of heat stress resilience in dairy cattle

**DOI:** 10.1371/journal.pone.0206520

**Published:** 2018-11-01

**Authors:** Elena Galán, Pol Llonch, Arantxa Villagrá, Harel Levit, Severino Pinto, Agustín del Prado

**Affiliations:** 1 Basque Centre for Climate Change (BC3), Leioa, Spain; 2 Departament of Animal and Food Science, Universitat Autònoma de Barcelona, Barcelona, Bellaterra (UAB), Spain; 3 Centro de Investigación en Tecnología Animal (CITA), Valencian Institute for Agricultura Research (IVIA), Segorbe, Spain; 4 Institute of Agricultural Engineering, Agricultural Research Orgazation (ARO)- Volcani Center, Bet Dagan, Israel; 5 Engineering for Livestock Management, Leibniz Institute for Agricultural Engineering and Bioeconomy (ATB), Potsdam, Germany; University of Illinois, UNITED STATES

## Abstract

**Introduction:**

Projected temperature rise in the upcoming years due to climate change has increased interest in studying the effects of heat stress in dairy cows. Environmental indices are commonly used for detecting heat stress, but have been used mainly in studies focused on the productivity-related effects of heat stress. The welfare approach involves identifying physiological and behavioural measurements so as to start heat stress mitigation protocols before the appearance of impending severe health or production issues. Therefore, there is growing interest in studying the effects of heat stress on welfare. This systematic review seeks to summarise the animal-based responses to heat stress (physiological and behavioural, excluding productivity) that have been used in scientific literature.

**Methods:**

Using systematic review guidelines set by PRISMA, research articles were identified, screened and summarised based on inclusion criteria for physiology and behaviour, excluding productivity, for animal-based resilience indicators. 129 published articles were reviewed to determine which animal-based indicators for heat stress were most frequently used in dairy cows.

**Results:**

The articles considered report at least 212 different animal-based indicators that can be aggregated into body temperature, feeding, physiological response, resting, drinking, grazing and pasture-related behaviour, reactions to heat management and others. The most common physiological animal-based indicators are rectal temperature, respiration rate and dry matter intake, while the most common behavioural indicators are time spent lying, standing and feeding.

**Conclusion:**

Although body temperature and respiration rate are the animal-based indicators most frequently used to assess heat stress in dairy cattle, when choosing an animal-based indicator for detecting heat stress using scientific literature to establish thresholds, characteristics that influence the scale of the response and the definition of heat stress must be taken into account, e.g. breed, lactation stage, milk yield, system type, climate region, bedding type, diet and cooling management strategies.

## 1. Introduction

There has been growing interest in studying the effects of heat stress in dairy cows as this is a welfare and production problem in temperate latitudes which is expected to increase in the near future. According to recent predictions [1], global temperatures are expected to rise by 1.4–3.0°C by the end of this century, and by 5.0°C in certain temperate areas of the planet. An average temperature rise of 2°C or more is also expected in the upcoming years, along with an increase in the frequency and intensity of extreme heat waves.

Heat stress has an impact on a variety of parameters in dairy production, particularly in terms of reductions in milk production, impaired reproductive performance, higher risk of lameness and mastitis and even death [2]. Numerous studies have focused on the productivity-related effects of heat stress in lactating cows [3], dry cows [4] and calves [5]; and on fertility effects [6,7]. However, interest has recently turned to studying the effects of heat stress on welfare [8–10], which may or may not impact production or reproductive parameters. Some of the welfare effects based on physiological and behavioural changes that result from heat stress may occur in the short term, sometimes with no impact on productivity [10], while others can predict further impacts on productivity and therefore be used to prevent them [11].

The Temperature Humidity Index (THI) is the most widely used environmental indicator of heat stress effects in scientific literature. The heat stress threshold has been set at daily THI values of 68–72 [8,12,13], although lower values have recently been found for temperate areas [14–16]. In addition, in outdoor systems animals are exposed to sunlight, which increases the effects of heat stress [17,18], and to high winds, which decreases them [19]. These parameters are not taken into account in THI. In animal welfare, changes in behaviour or physiology are referred to as animal-based welfare measures, since they reflect the state of an animal in a given environment or challenge. Animal-based indicators (ABI) can be used to recognise heat stress more accurately than environmental indicators as they take into account differences between individuals and differences in farm systems (e.g. pasture or not, outdoor access or not) which can be used to assist in heat stress prevention and mitigation and improve the welfare of dairy cows.

Resilience is the ability of animals to adapt to short-term environmental challenges [20]. In dairy cattle, resilience to heat stress refers to the animals’ response to a heat challenge in terms of maintaining thermal balance and recovering homeostasis. This study focuses on the animal-based indicators of resilience to a heat challenge in dairy cattle. Animal-based indicators can be locomotive (e.g. standing to expose more surface area to air flows), physiological (e.g. increased panting rate, sweating, drooling) or feeding behaviour (e.g. reduced feed intake) [21,22]. All these changes share one main common goal: to maintain body temperature by either decreasing metabolic heat production or losing heat by conduction, convection, radiation and water evaporation. The failure of these mechanisms leads to a body temperature rise and the associated heat stress response [10].

The aim of this study is to identify the animal-based indicators of heat stress (excluding productivity-related measures) that have been most widely used in scientific literature for measuring resilience in dairy cows. The outcome of this study will help refine the identification of heat stress in dairy cattle, which may in turn help to mitigate its effects on productivity and welfare.

## 2. Materials and methods

### 2.1 Search criteria and strategy

Peer-reviewed scientific literature published before 24^th^ February 2017 was systematically reviewed. Searches were performed using the same search items on three search engines: (1) PubMed; (2) ScienceDirect; and (3) Scopus. The search terms used (including titles, abstracts and keywords) were ("heat stress*" OR "heat load" OR "temperature humidity index" OR hyperthermia OR "thermal stress*") AND (cow* OR cattle OR heifer* OR "lactating cow*" OR "dry cow*" OR "bos taurus") AND dairy AND (physiolog* OR behavi*r OR welfare OR health). PubMed does not accept truncation in the middle of words, so behavior OR behaviour was used.

**Fig 1:**
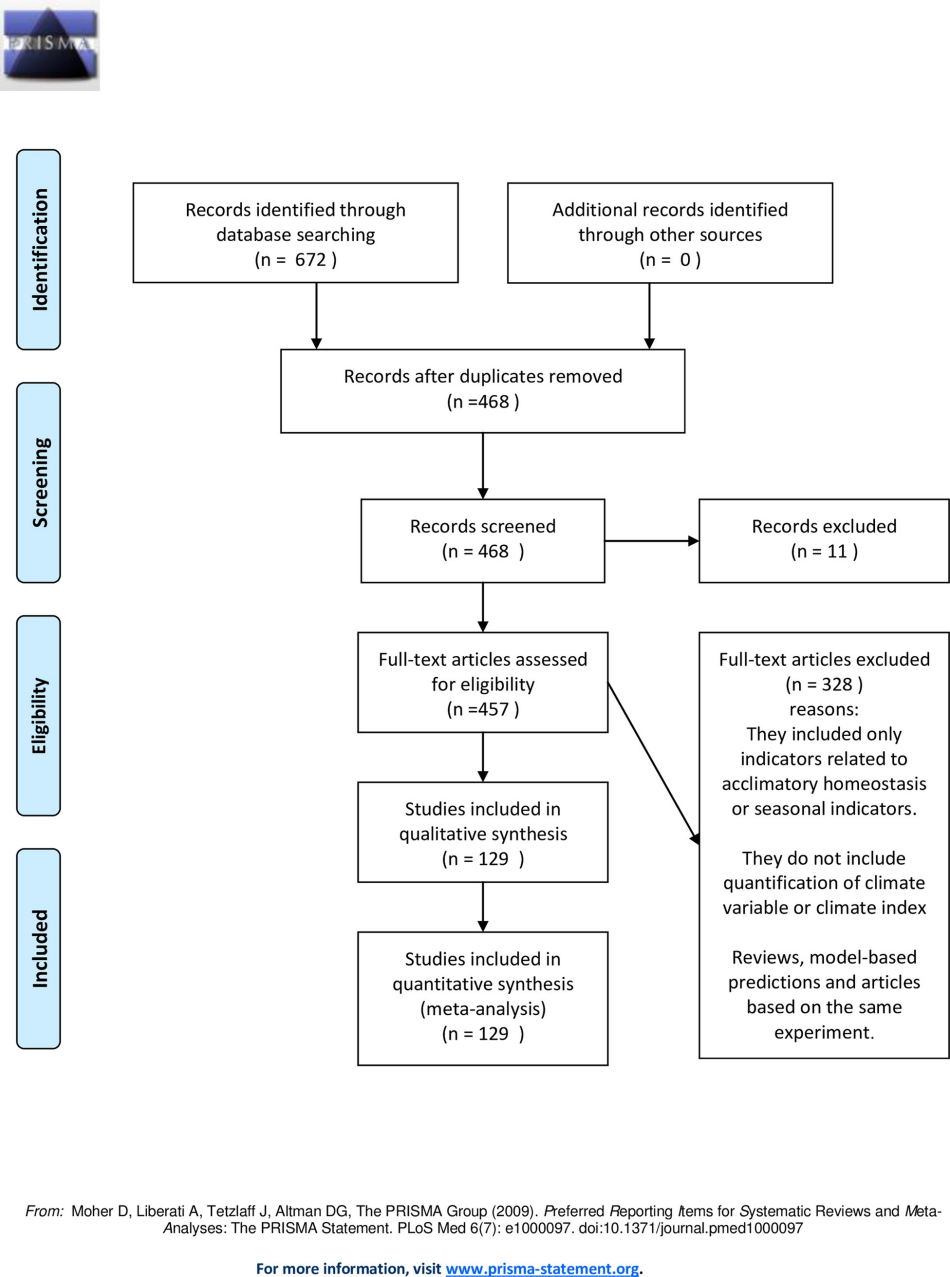
PRISMA 2009 flow diagram.

Most studies describing a single average value for an entire warm season did not discriminate between short- and long-term acclimation or include resilience among their keywords, so we designed a broader question excluding keywords such as resilience and acclimation and made our selection in a second step. After filtering by documents written in English, journal articles and proceedings, 672 documents were obtained (108 in (1) PubMed, 118 in (2) ScienceDirect and 446 in (3) Scopus). The number fell to 468 when duplicates were removed. Eleven of the 468 full text papers could not be obtained online access or by contact with the authors. From the information in the abstracts, we know that 4 of the 11 would not meet the study selection criteria below (see Fig 1). The PRISMA checklist is in S1 Table.

### 2.2 Study selection criteria

We selected experimental studies that related climate variables (e.g. temperature) or indices (THI, BGHI and others such as Adjusted THI [23], Back Globe Humidity Index (BGHI) [18], Heat Load Index (HLI) [24], Comprehensive Climate Index (CCI) [25]) to physiological and behavioural responses associated with thermoregulation. We therefore excluded changes in endocrine status, e.g. growth hormones or glucocorticoid levels [26,27].

Similarly, effects on somatic cell count (SCC) were excluded as they result from seasonal (and thus long-term) warm temperatures among other factors, such as increased exposure to pathogens or vectors [28,29]. In addition, we removed reviews, model-based predictions and articles based on the same experiment. Once all these selection criteria were applied, 129 articles remained. These articles are listed in S1 List.

Apart from quantifying the list of ABIs, we included information on characteristics of the studies that were likely to be relevant to users of the review. These characteristics were defined prior to the literature search [30] and were grouped into characteristics that could influence the definition of heat stress and the scale of the effect. An examination of these characteristics (S2 Table) demonstrated that the studies selected did not all use the same methodology, so we discarded the meta-analysis.

## 3. Results and discussion

### 3.1. The database

The database for the systematic review comprises a list of all the animal-based indicators used in the studies to measure heat stress. The last two subsections list those characteristics of the studies that may influence the scale of heat stress and the definition of heat stress. These characteristics are listed in S2 Table.

#### 3.1.1. List of the animal-based indicators (ABI) identified for measuring heat stress

**Fig 2:**
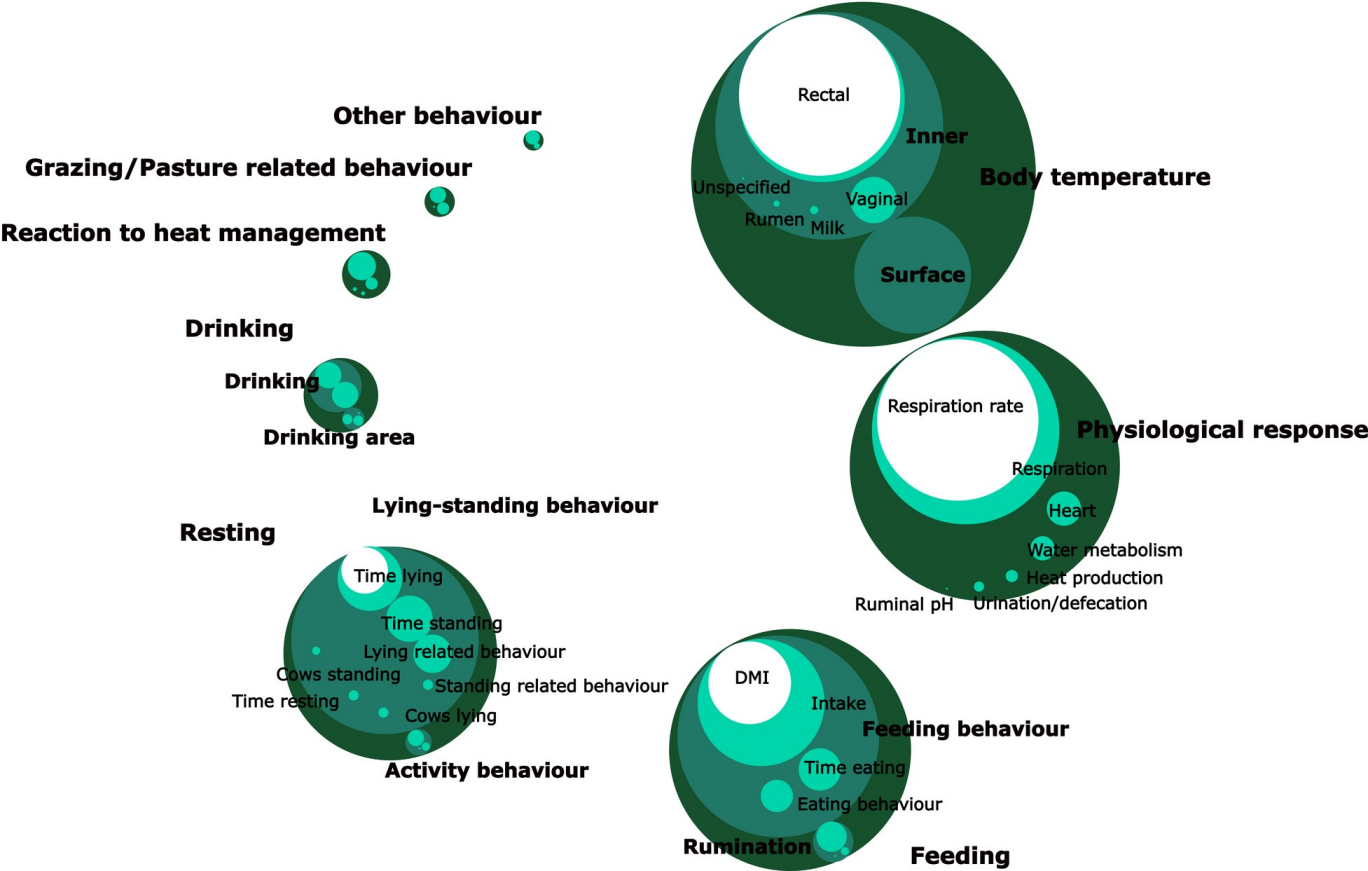
Animal-based indicators (ABIs) of heat stress of dairy cows. Circle packing plot representing showing how many studies use the most widely used ABIs listed in Table 1 and S3 Table. The diameter of the circles is proportional to the number of occurrences of each ABI included in each aggregation level (bold). The darker the circle, the higher the level of aggregation.

The most frequent animal-based indicators of heat stress are shown in Table 1 and Fig 2. Indicators related to internal temperature (used in 28% of the studies) are the most common group, and rectal temperature is the predominant measure among them, followed by vaginal and milk temperatures respectively. We separated body temperature from other physiological indicators because the latter refer to physiological organic strategies to reduce body temperature. The rectal temperature of dairy cows is normally around 38.5°C and thresholds for fever range from 39.4 to 39.7°C [31]. On average, vaginal temperature is 0.2± 0.3°C higher than rectal temperature [31]. According to Reference [32], the average milk temperature of dairy cows is 0.16°C higher than their rectal temperature.

**Table 1:** Summary of the most frequent indicators used to assess heat stress in dairy cattle.

Animal based indicator	% over total studies (n = 129)
**Body temperature**	32.1
**Inner temperature**	27.6
**Surface temperature**	6.4
**Physiological response**	26.2
**Feeding**	16.8
**Feeding behaviour**	14.1
**Rumination time**	3.2
**Resting**	9.4
**Lying-Standing**	8.2
**Activity behaviour**	2.7
**Drinking**	7.6
**Drinking**	5.8
**Drinking area**	1.3
**Grazing/Pasture related behaviour**	3.2
**Reaction to heat management**	2.9
**Other behaviour**	1.8

The rows coincide with the circles in Fig 2. The full list of animal-based indicators is in S3 Table. The percentages of the subcategories may not add up because the same study may include indicators belonging to different subcategories of the same category. In such cases, they count as a single study for the aggregate category to avoid double counting.

The second most common group is that of physiological indicators (24% of the studies). In it, the most prominent variables are respiration rate (also panting), heart rate, sweat rate and metabolic heat production. Respiration rate increases as THI increases: for instance a respiration rate higher than 60 breaths per minute is considered an indicator of heat stress in lactating dairy cows [27]. The sweating rate has its own cyclical pattern and does not follow increases in THI linearly, but it is higher in hot, dry conditions than in hot, humid conditions [33]. In heat-stressed lactating cows, water excreted in urine increases and faecal water decreases, but these changes are not confirmed in dry cows [34]. In summer-reared calves, [35] finds more urination and defecating bouts. There is no clear pattern for heart rate: some studies show an increase [36–38] and others a decrease [34,39,40] as a result of heat stress. Adding to these inconclusive results, [10] observe both an increase and a decrease in heart rate as a response to heat stress. Alternatively, [3] states that heat stress initially increases respiration rate and heart rate but that they tend to slow down later on.

Feeding behaviour is used to assess heat stress in 14% of the studies. The most frequent variables are dry matter intake (DMI), feeding time and ruminating time. Reducing feed intake is an adaptive mechanism for cows to reduce metabolic heat production [3,27] and can be detected within two days after the onset of the heat challenge [32,36]. A decrease in feed intake is accompanied by shorter meal duration [39,41,42] and decreased feeding time [35,39,41–46]. The frequency of meals per day increases during heat stress in high-yield or late-gestation cows [41,42] whereas it decreases in those with lower yields [39] and in postpartum cows [41]. In fact, high-yielding dairy cows have higher metabolic heat production and are therefore more sensitive to heat stress [3]. Rumination time also decreases in the event of heat stress [3,46]; however there is also a lower passage rate, which can increase rumination time per feed intake [47].

Resting-associated measures appear in 9% of the studies on heat stress considered, with lying time and standing time being the most frequent of them. In general warm temperatures decrease the time spent lying. This behaviour has been explained by the fact that a standing cow exposes more surface area to convection and therefore loses more heat than a lying cow [22,48]. Cows in thermal comfort have been reported to lie for approximately 70 min [48], and to prefer to lie down for about half their time [19,49] or up to 14 h/day for the most productive among them [50]. Standing time does not increase linearly with THI: it peaks at THI 80 to 89 rather than THI 90 to 98 or THI >98. This is probably related to the tiredness caused by the accumulated time spent standing while THI is rising [48].

Drinking-related indicators are studied by 8% of the studies that assess heat stress. Water intake increases by 1.2 kg per°C of increase in minimum ambient temperature [51],. Proximity to the water trough increases when there are no sprinklers or shade available to mitigate heat [52,53]. Heat-stressed cows drink more frequently [35,41,52,54] but take in smaller quantities of water and the total time spent drinking increases [35,44,46,55].

6% of the studies considered measure cow surface temperature, which increases with high ambient temperatures although the amount depends on where on the body it is measured and on whether the area measured is shaved [56]. Shorter hair, e.g. on Holstein cows with slick genes, makes for better inner temperature regulation [57]. In addition, peripheral areas such as the thigh and the leg are more affected by THI than the surface temperature measured on the udder [58]. Skin temperature does not always reflect changes in inner temperature [59]. Thus, inner temperature seems to be a more valuable parameter for assessing heat stress, but it is not easy to measure in a free-range animal [10].

Grazing or pasture-related behaviour is measured in 3% of the studies. Time spent grazing decreases with heat stress [52,55]. For instance, in experiments where cows can choose between being on pasture or indoors, they prefer to stay indoors in events of higher THI or BGHI [60,61], although this trend is not so clear when THI is in the range of thermal comfort [62–65]. When given the choice, cows also prefer to be on pasture at night, thus avoiding diurnal THI peaks [60,64,65]. Finally, winds can help mitigate heat stress in animals on pasture even if the cows are exposed to strong solar radiation [19].

Heat stress abatement strategies other than those already mentioned are covered by 3% of the studies considered. They include time in the shade, time under sprinklers, signs of comfort or discomfort related to water flow, etc. At high temperatures, the use of fans or fans with misters in feeding areas lead cows to spend longer there than if no cooling measure are taken, although total intake might not be affected [43,53,66,67]. The use of shade is also a common strategy for mitigating heat stress, and the higher the solar radiation the more time cows spend in the shade [68]. When given the choice, cows spend more and more time under sprinklers as the ambient temperature rises [69], but they are reluctant to expose their heads to the water [43,53,70]. An example of this is found in [71], where there is evidence that sprinkler treatment reduces the number of expressions of discomfort such as hoof stamps or tail flicks.

Other variables that cannot be classified within the categories listed above are covered in 2% of the studies. These include aggressive behaviour, which increases during heat stress in the absence of shade [52], body positions to increase wind exposure vs “normal” body positions [19], and grooming, which decreases under heat stress [35].

Taking the individual variables from all categories, rectal temperature (12.4%), respiration rate (12.4%), dry matter intake (6.4%), time lying down (3.8%) and vaginal temperature (3.0%) are the indicators most frequently used to assess heat stress in dairy cattle. The full list of indicators reported within each category is shown in S3 Table.

#### 3.1.2 Characteristics that influence the scale of heat stress

Here we provide information on the characteristics that may influence the scale of ABIs. Studies are classified according to production systems (indoor, outdoor and grazing), breed, milk yield (genetic merit) and production stage (e.g. lactating or dry).

11% of the studies do not give enough data to identify the system, but 45.5% have some outdoor access (16% pasture, 29.5% no pasture), 29.5% are indoor and 14% use a thermal chamber).

**Fig 3:**
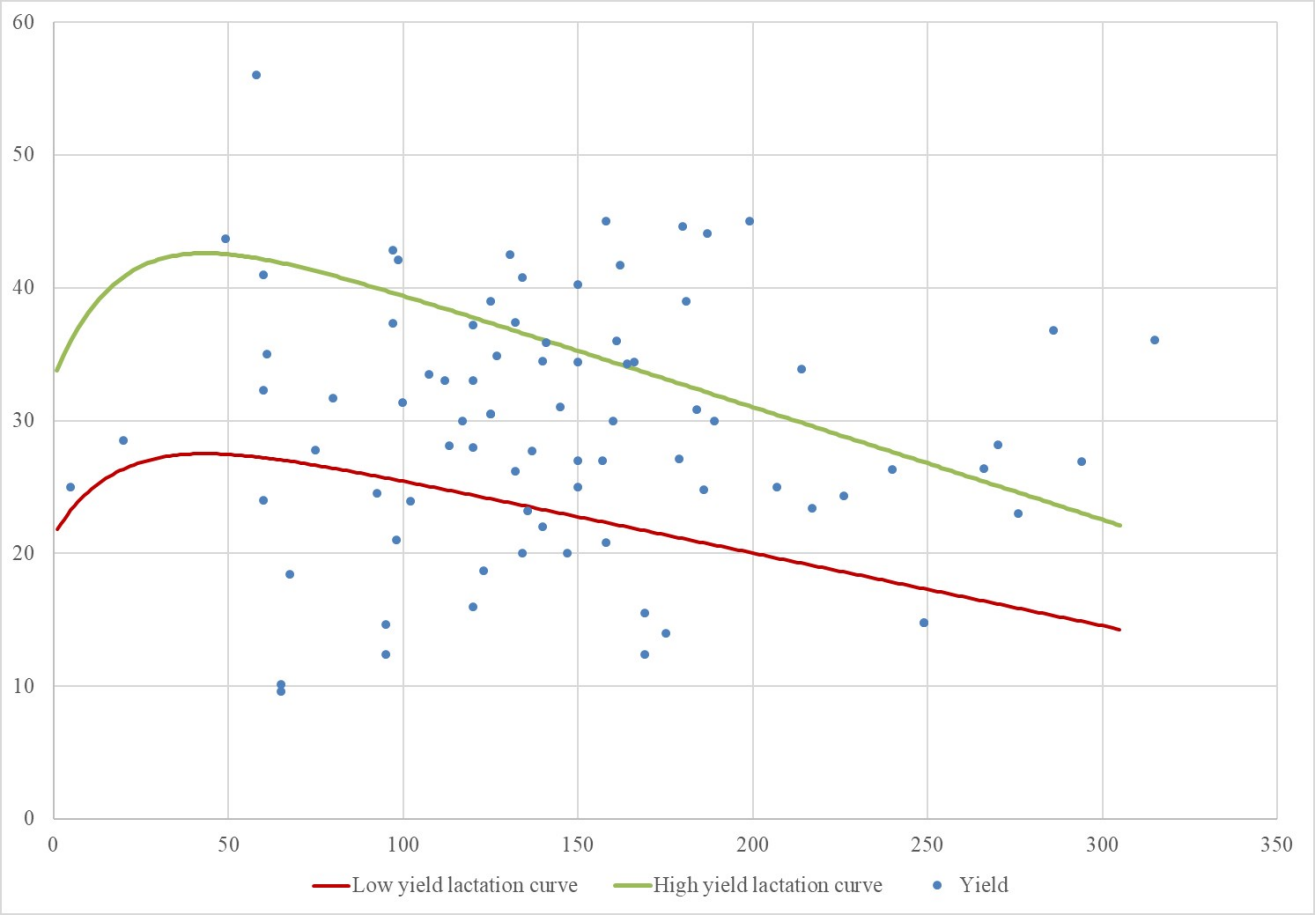
Classification of yields when DIM are reported. Source: own work from the milk yields and days in milk reported in the studies. From the red line downwards the yields are <22 kg/d using standardised lactations of 305 days. From the green line upwards the yields are >34 kg/d.

The studies focus mostly on Holstein breed cows (78%) in lactation (81%), although some studies use dry cows (11%) or calves and heifers (4%). Most of the lactating cows are in mid-lactation (65–225 days in milk). There is inconsistency between the studies in considering a milk yield average as “low” or “high”. For instance, for the same breed [46] considers a milk production of <20 kg/d as “low” and >25 kg/d as “high”, whereas [39] considers a winter milk yield of 28kg/d as “low” and 33 kg/d as “high”. Therefore, to compare milk yields within the studies, we take all the annual averages reported, calculate the first and third quartiles, i.e. ≤22 kg/d and ≥34 kg/d, and define them as “low” and “high” respectively. On that basis, we calculate the corresponding “low” and “high” lactation curves using the SIMSdairy model [72] (Fig 3). We plot the yield values in relation to the days in milk reported at the beginning of those studies that do not report annual average production. Under our classification, 18% of the studies work with low yields, 29% with medium, 30% with high and 23% with yields which are either not defined or provide insufficient data for them to be classified. The yield levels are unevenly distributed across the systems, but the system with the highest proportion of low-yielding cows (33%) is the pasture system, and that with the highest proportion of high-yielding cows (34%) in indoors.

According to the Köppen climate classification [73], 57% of the studies considered were conducted in temperate climates (34% in temperate climates with warmest month average >22°C (humid subtropical (Cfb) and Mediterranean (Csa)) and 22% in temperate climates with warmest month average <22°C (oceanic (Cfb), Mediterranean with warm summer (Csb) and highlands (Cwb)). 18% were conducted in arid climates, 14% in continental climates and 10% in tropical climates. The rest are unspecified.

There is no way of knowing whether our studies are representative of current farms, but the studies conducted in arid climates do not include pasture. In temperate climate studies, the systems are evenly distributed between indoor, outdoor without pasture and pasture, but the use of thermal chamber is marginal (8%). By contrast, in arid and continental climates the use of a thermal chamber is more widespread at 26% and 28% respectively. In arid climates the most widely studied treatment for mitigating heat stress is that of cooling systems (48% of all studies in arid climates) installed in barns, while in temperate climates the treatments studied are more diverse: 48% of the studies include treatments (diets, shade, supplements, access to pasture and others) other than cooling systems within barns (which are used exclusively in just 26% of cases). In tropical systems barn cooling systems are marginal (8%) and the focus is on other treatments (46%) or on measuring heat stress effects *per se* (46%).

70% of the studies conducted in tropical climates include breeds other than Holsteins, but in all other climates more than 80% of the studies use only Holsteins.

#### 3.1.3 Characteristics that influence the definition of heat stress

This section addresses the design of experiments for studies to evaluate heat stress. That design influences the definition of heat stress used by each study. The characteristics selected are system type, environment comparison, use of heat mitigation measures, climate indices for measuring heat load and heat stress duration.

The controlled environment of the thermal chamber enables heat stress to be compared with thermo-neutral control conditions (13%), except in a single study that uses air conditioning, enabling the temperature of the barn to be changed [74]. This effect is emulated in the other systems by comparing a cool period with a warm period (13%), for example different seasons. However, most of the studies considered (60%) have a single group exposed to heat stress with no control group (a thermo-neutral environment).

68% of the studies include the study of a heat stress mitigation treatment. 11% of them use a thermo-neutral control environment, 6% use a cooler season as a control group and 73% make their measurements in a warm period or season. By contrast, 31% focus on studying the harmful effects of heat stress with no mitigation measures. Only 17% of them use a thermo-neutral control environment, 29% use a cooler season as a control group and 31% make their measurements in a warm period or season.

In the studies investigating management to mitigate heat stress, the most widely used treatments are cooling systems (29%), a special diet or supplement (29%), shade (11%), pasture restriction or preference (8%) and others (10%), which include bedded pack systema, bedding, feeding method and drinking water temperature.

86% of the studies use the THI (maximum, average, minimum) or temperature and humidity together as a measure of climate, while 13% use temperature without calculating the THI. 36% of the studies also factor in solar radiation, wind speed or other indices that include them (e.g. the Black Globe Humidity Index (BGHI), Heat Load Index (HLI), adjusted THI, Heat Load Index (HLI) or Comprehensive Climate Index (CCI)). These indices are used especially in studies of pasture systems (43%, or 66% if studies that include rainfall are also considered). Studies measuring heat stress effects on behaviour and physiology in pasture find closer correlations with climate parameters if the effect of solar radiation is added, i.e. if black globe temperatures [18,24,75,76] are also used rather than THI only. This is important as shade is one of the cooling strategies most widely studied in pasture systems. For systems other than pasture, the proportion of studies that include additional climate variables or indices other than THI is only 20%, even when cows have access to outdoors (i.e. playground but not pasture) and are therefore exposed to solar radiation or wind.

Duration of heat stress depends on each study’s own definition of a heat stress threshold (e.g. days with daily average THI>72), or in the absence of such a definition on the period when effects of heat stress are detected. If the periods of heat stress correspond to different trials (with different treatments or just replications) we consider a single trial period (or take the average if they have different durations) and not the entire experiment. When studies show a relationship between THI and any ABI but do not show THI measurements, we consider the number of days of exposure to a hot environment as unknown. We realise that this is a complex categorisation that might be subject to interpretation bias, so we prefer to express this information qualitatively, thinking that readers may be interested in locating studies according to broad guidelines of duration (short, medium or longer) of exposure to heat stress.

In 34% of the studies considered the duration of heat stress is less than one week, and most of these studies are conducted under chamber conditions, as are the 17% that last from one to three weeks. 53% of the studies have periods of heat stress longer than three weeks (>21 days); these are mostly those classed as “other” studies using systems other than climatic chambers.

### 3.2 Factors that condition the response of animal-based indicators as measures of heat stress

All the responses to ABIs identified can be influenced by a variety of internal (e.g. breed, metabolic status) and external factors (e.g. system type, heat stress duration). In this section we discuss the influence of different factors in the assessment of heat-stress response as result of the systematic review.

#### 3.2.1 Genetic and phenotypic background

Holstein cows tend to have higher internal temperatures under hot conditions [32,52,77,78] than other breeds (e.g. Jersey, Brown Swiss) and crossbreeds.

Holstein cows are usually larger and have thicker coats than other dairy cattle breeds, which hinders heat loss [79]. Some characteristics of other breeds such as short hair, light colour, low body weight, high rate of cutaneous evaporation, low milk production (and therefore low metabolic heat production) benefit the heat stress tolerance of other breeds [78,80].

White cows have also lower skin temperatures than black cows under high solar loads, which compromises the flow of metabolic heat towards the surface to be lost by convection or evaporation. Black cows exposed to solar radiation therefore have a greater risk of thermal discomfort [19,33].

The effects of heat stress are more severe in high-yield dairy cows than low-yield cows, as higher yields are related to higher feed intakes, which in turn is linked to greater metabolic heat [81]. Holstein is the main breed in high-yield dairy cattle, so severe effects of heat stress are more frequent in this breed due to its association with milk yield potential [39,46].

#### 3.2.2 Metabolic status

Heat stress sensitivity can be determined by the metabolic status of cows, as this has an impact on heat production. In cattle, the main metabolic statuses that can alter metabolic heat production are lactation and gestation.

Lactating cows have more heat to dissipate than non-lactating cows and also have greater difficulty dissipating heat during hot, humid conditions [51]. On the other hand, dry cows also suffer from heat stress, which might compromise subsequent lactation, as heat-stressed dry cows in the late stages of pregnancy show lesser development of mammary glands than those in barns with heat abatement structures [4]. As milk yield declines with the stage of lactation, it is reasonable to think that sensitivity to heat stress may also decrease. However, a study by [82] finds that body temperature, respiration rate and milk yield are more severely affected in mid-lactation (80–120 days in milk) cows than in the early or late stages. The reason for this inconsistency could be that in early lactation, milk production is supported by stored tissue mobilisation and less by feed intake, by contrast with the mid-lactation stage, when peak intake occurs. Therefore, early-lactation cows produce less metabolic heat per kg of milk [83].

Despite the fact that heifers are less sensitive to heat stress than lactating cows [84], it is difficult to tell from the articles reviewed whether pregnant cows are more or less sensitive to heat stress than non-pregnant cows. Calves exposed to heat stress *in utero* have higher rectal temperatures and respiration rates and longer lying times [85], but they could be subject to acclimation in the long run and therefore be less sensitive to heat stress. Once raised, lactating cows from heat-stressed dams have smaller increases in rectal temperature and sweating rate than their peers from cooled dams when exposed to heat stress [86].

#### 3.2.3 Heat challenge duration

Circadian rhythms influence response to heat load, as body temperatures are lower in the morning than in the afternoon [87]. The circadian rhythm affects the lying time of cows, i.e. cows tend to lie more at night [88]. Also, temperature drops at night and night cooling below the traditional 72 THI threshold alleviate the effects of heat stress. However, this threshold is set by using milk yield and mortality risk as indicators [2] and it might be different if other indicators are used. For instance, night cooling (around 67THI or lower) does not affect diurnal respiration and heart rate when cows are exposed to THI above 75 [52] with no shade. When the experiments are conducted in thermal chambers, the adoption of a cyclic pattern of temperatures to simulate night cooling (around 66THI) reduces the severity of heat stress in rectal temperature and milk yield [89].

The duration of heat stress and the acclimation capacity of each cow affects the value of the response. For example, rectal temperature, respiration and heart rates are observed to increase during the early days of exposure but then to drop progressively [11,90,91]. This may be due to the acclimation process, which reduces endogenous heat production and facilitates heat loss [91]. Similarly, the fall in dry matter intake is less severe after three weeks of warm temperatures, suggesting that cows start to acclimate [91,92]. The acclimation process involves changes in endocrine status which lead to homeostasis if exposure to thermal load is prolonged (and not lethal). How long it takes to reach this new equilibrium also varies from breed to breed, e.g. it ranges from 9 days for Angus and Charolais to 14 days for Polled Hereford [26]. Further studies are needed to predict the effects under long-term or cycling heat-stress conditions [11].

#### 3.2.4 System type and management

The term “cooling systems” refers to different designs of fans, sometimes in combination with misters or sprinklers which are installed in barns. However, they have to be adapted to farm needs as they are not always sufficient to alleviate heat stress. For instance, decline in milk yield is found to be at a higher threshold (83.2 THI) with a combination of a shaded pen close to the milking room plus water sprinkling throughout the day than otherwise [45]. The frequency of cooling is also determinant for the effect: for instance, when it is increased from 5 to 8 times per day in an environment of 78.4 THI average for 40 days, cows show lower respiration rates and rectal temperatures and spend more time resting [93].

Orientation of fans and misters is also important for how effective they are. [94] demonstrate that if fans and misters can follow shade as the sun’s angle shifts throughout the day, lower body temperatures and more time lying (80.2 THI during 5 days) are obtained than with a fixed fan and mister system. In grazing systems, if sprinkler treatments are introduced before milking, rectal temperature is lower at a daily average THI for a week of between 73.8 and 77.9 [95]. Although the use of sprinklers gives good results, it has raised environmental and economic concerns because it involves a large increase in water consumption at dairy farms [8]. Diet also has an impact on the response of dairy cows to heat stress: overall its effects on saving metabolic heat production by supplementing saturated fatty acids [96,97], reducing neutral detergent fibre on a dry matter basis [98–100] and increasing the proportion of digestive fibre [101] have been studied. Indeed, replacing low quality NDF in diets by NDF of better quality (low lignin concentrations and high digestibility) modifies the effects of heat stress on performance, body temperature, rumination activity and feeding behaviour [100,102,103]. The above strategies must be applied with caution as they can increase the risk of rumen acidosis in reducing fibre. Reference [104] suggests that these strategies need to be studied further. In addition to diets, several attempts to minimise the effects of heat stress throug supplements have been made. These supplements include citrus extract [105], niacin [56,106], conjugated linoleic acids [107], Asparagus officinalis [108] and yeast cultures [109], but most of them have inconsistent effects on the responses of animals to heat stress and further research is needed to understand the real extent of their effects [104].

#### 3.2.5 Underlying factors that affect resting behaviour

There are other factors that affect resting behaviour as an indicator of heat stress. Lying down facilitates body heat loss by convection, so wind speed may affect the convection rate and therefore also affect standing time in heat-stressed cows [19].

A key factor for determining resting behaviour is bedding quality, which affects comfort, including thermal comfort. Some studies investigate how heat stress changes preferences for lying on different bedding substrates. With higher THI cows tend to prefer substrates that dissipate body heat better and allow for evaporation, such as wood shavings and solid manure, rather than ethylene-vinyl acetate **(**EVA) mats [110] and sand rather than straw bedding [111].

The activities of cows on pasture differ from those of confined cows. Resources such as water and feed might need longer displacements in pasture, so they may spend less time lying down than confined cows [110]. Also, grazing and the fact that bedding on pasture may facilitate the transition from lying to standing so make for longer standing times [60].

Shorter resting time is a reaction to high temperatures, but it is associated with the risk of claw-horn lesions and could be related to the higher number of cows which are lame (or show some degree of lameness) observed at the end of summer [48,112]. In turn, lameness alters preferences and standing-lying behaviour in relation to bedding material, for instance there is no difference between non-lamed cows but lame cows spend more time standing on rubber-crumb mattress surfaces than on sand bedding. This suggests that they prefer more cushioned surfaces to stand on as they struggle to change position on mattress stalls [113]. Therefore, lying time is also influenced by factors other than thermal comfort, which evidences the need to use this measure in combination with other indicators.

## 4. Implications for the dairy sector

We have identified 129 papers with 212 different animal-based indicators of heat stress. Those most widely used concern temperature, physiology, feeding and resting behaviour. These indicators can be used to identify animal-based responses to stress from high temperatures and humidity. The advantage of animal-based indicators is that they enable individual sensitivity to heat stress to be estimated with precision. At the same time, using animal-based indicators helps to determine which measures of heat mitigation are most efficient when the predictive value of THI alone is limited, e.g. with direct sunlight or inside barns if the THI measurement is taken outdoors. Body temperature and respiration rate are the indicators in all categories which are most frequently used to assess heat stress in dairy cattle. The most widely used behavioural indicators are time lying, time standing and time eating. In terms of individual variables from all categories, rectal temperature, respiration rate, dry matter intake, time lying and vaginal temperature are the indicators most frequently used to assess heat stress in dairy cattle.

The studies considered in this review have characteristics that must be taken into account in estimating individual heat stress responses. Hence, when choosing an ABI to detect heat stress and using scientific literature to establish thresholds, we recommend taking into account that most ABIs refer to lactating Holstein cows which produce more than 34 kg/d. In addition, the experiments comparing a thermo-neutral environment with a heat stress environment are conducted using thermal chambers. Under other farm environments (indoor, outdoor, pasture) factors may intervene which are different from those found in thermal chambers, but may be similar to real conditions. Those ABIs associated with resting behaviour are influenced by a number of management factors such as bedding type, tiredness and presence of pasture, among others. The duration of exposure to hot conditions should also be taken into account so as to capture the effects of acclimation.

Moreover, the temperature ranges and environmental indices considered are not standardised across the studies reviewed, so we recommend taking into account the climate of the region, for instance using the Köppen climate classification. Further research is needed to determine the limitations and possibilities of climate change adaptation strategies.

## Supporting information

S1 Table PRISMA 2009 checklist. (DOC)

S2 Table Description of the factors of the studies found in the systematic review. (XLSX)

S3 Table ABI indicators found in the systematic review. (XLSX)

S1 List Systematic review references list. (DOCX)
