# Supplementary material for: A systematic review of non-productivity-related animal-based indicators of heat stress resilience in dairy cattle
Source: PLoS One. 2018 Nov 1;13(11):e0206520. doi: 10.1371/journal.pone.0206520 (PMC6211699; doi:10.1371/journal.pone.0206520)
Supplement: S1 List — (DOCX) [file pone.0206520.s004.docx]

**S2 List. Systematic review references list**

Abeni F, Calamari L, Stefanini L. Metabolic conditions of lactating Friesian cows during the hot season in the Po valley. 1. Blood indicators of heat stress. International Journal of Biometeorology. 2007;52(2):87–96.

Alfonzo EPM, Barbosa da Silva MVG, dos Santos Daltro D, Stumpf MT, Dalcin VC, Kolling G, et al. Relationship between physical attributes and heat stress in dairy cattle from different genetic groups. International Journal of Biometeorology. febrero de 2016;60(2):245–253.

Allen JD, Hall LW, Collier RJ, Smith JF. Effect of core body temperature, time of day, and climate conditions on behavioral patterns of lactating dairy cows experiencing mild to moderate heat stress. Journal of Dairy Science. 2015;98(1):118–127.

Anderson SD, Bradford BJ, Harner JP, Tucker CB, Choi CY, Allen JD, et al. Effects of adjustable and stationary fans with misters on core body temperature and lying behavior of lactating dairy cows in a semiarid climate. Journal of Dairy Science. 2013;96(7):4738–4750.

Arieli A, Adin G, Bruckental I. The effect of protein intake on performance of cows in hot environmental temperatures. Journal of Dairy Science. 2004;87(3):620–629.

Arieli A, Rubinstein A, Moallem U, Aharoni Y, Halachmi I. The effect of fiber characteristics on thermoregulatory responses and feeding behavior of heat stressed cows. Journal of Thermal Biology. 2004;29(7–8):749–751.

Avendaño-Reyes L, Alvarez-Valenzuela F, Correa-Calderón A, Fadel J, Robinson P. Is soaking cows during dry period an effective management tool to reduce heat stress and improve pospartum productivity ? Journal of Applied Animal Research. 2008;34(1):97–100.

Avendaño-Reyes L, Hernández-Rivera JA, Álvarez-Valenzuela FD, Macías-Cruz U, Díaz-Molina R, Correa-Calderón A, et al. Physiological and productive responses of multiparous lactating Holstein cows exposed to short-term cooling during severe summer conditions in an arid region of Mexico. International Journal of Biometeorology. 2012;56(6):993–999.

Berman A. Forced heat loss from body surface reduces heat flow to body surface. Journal of Dairy Science. 2010;93(1):242–248.

Bewley JM, Grott MW, Einstein ME, Schutz MM. Impact of intake water temperatures on reticular temperatures of lactating dairy cows. Journal of Dairy Science. 2008;91(10):3880–3887.

Black RA, Krawczel PD. A case study of behaviour and performance of confined or pastured cows during the dry period. Animals. 2016;6(7).

Boga M, Gorgulu M, Sahin A. Effects of feeding methods, season and production level on lactation performance and fee ding behaviour of dairy cows. Bulgarian Journal of Agricultural Science. 2014;20(4):915–923.

Bouraoui RB, Ahmarb ML, Ajdoubc AM, Jemalic MD, Elyead RB. The relationship of temperature-humidity index with milk production of dairy cows in a Mediterranean climate. Animal Research. 2002;51:479–491.

Boyd J, West JW, Bernard JK, Block SS. Effects of plant extracts on milk yield and apparent efficiency of lactating dairy cows during hot weather 1. The Professional Animal Scientist. 2012;28(3):338–343.

Broucek J, Kisac P, Uhrincat M. Effect of hot temperatures on the hematological parameters, health and performance of calves. International Journal of Biometeorology. 2009;53(2):201–208.

Burfeind O, Suthar VS, Heuwieser W. Effect of heat stress on body temperature in healthy early postpartum dairy cows. Theriogenology. diciembre de 2012;78(9):2031–2038.

Calamari L, Petrera F, Stefanini L, Abeni F. Effects of different feeding time and frequency on metabolic conditions and milk production in heat-stressed dairy cows. International Journal of Biometeorology. 2013;57(5):785–796.

Calegari F, Calamari L, Frazzi E. Misting and fan cooling of the rest area in a dairy barn. International Journal of Biometeorology. 2012;56(2):287–295.

Chan S, Huber J, Chen K, Simas J, Wu Z. Effects of Ruminally Inert Fat and Evaporative Cooling on Dairy Cows in Hot Environmental Temperatures. Journal of Dairy Science. 1997;80(6):1172–1178.

Charlton GL, Rutter SM, East M, Sinclair LA. Effects of providing total mixed rations indoors and on pasture on the behavior of lactating dairy cattle and their preference to be indoors or on pasture. Journal of Dairy Science. 2011;94(8):3875–3884.

Charlton GL, Rutter SM, East M, Sinclair LA. Preference of dairy cows: Indoor cubicle housing with access to a total mixed ration vs. access to pasture. Applied Animal Behaviour Science. 2011;130(1–2):1–9.

Charlton GL, Rutter SM, East M, Sinclair LA. The motivation of dairy cows for access to pasture. Journal of Dairy Science. 2013;96(7):4387–4396.

Chen JM, Schütz KE, Tucker CB. Dairy cows use and prefer feed bunks fitted with sprinklers. Journal of Dairy Science. 2013;96(8):5035–5045.

Chen JM, Schütz KE, Tucker CB. Cooling cows efficiently with sprinklers: Physiological responses to water spray. Journal of Dairy Science. 2015;98(10):6925–6938.

Chen JM, Schütz KE, Tucker CB. Cooling cows efficiently with water spray: Behavioral, physiological, and production responses to sprinklers at the feed bunk. Journal of Dairy Science. 2016;99(6):4607–4618.

Cheng J, Zheng N, Sun X, Li S, Wang J, Zhang Y. Feeding rumen-protected gamma-aminobutyric acid enhances the immune response and antioxidant status of heat-stressed lactating dairy cows. Journal of Thermal Biology. 2016;60:103–108.

Cook NB, Mentink RL, Bennett TB, Burgi K. The effect of heat stress and lameness on time budgets of lactating dairy cows. Journal of dairy science. 2007;90(4):1674–1682.

Correa-Calderon A, Armstrong D, Ray D, DeNise S, Enns M, Howison C. Thermoregulatory responses of Holstein and Brown Swiss Heat-Stressed dairy cows to two different cooling systems. International Journal of Biometeorology. 2004;48(3):142–148.

da Costa ANL, Feitosa JV, Montezuma P.A. J, de Souza PT, de Araújo AA. Rectal temperatures, respiratory rates, production, and reproduction performances of crossbred Girolando cows under heat stress in northeastern Brazil. International Journal of Biometeorology. 2015;59(11):1647–1653.

da Cruz P, Monteiro C, Guimarães E, Antunes R, Nascimento M. Physiological parameters, hair coat morphological characteristics and temperature gradients in holsteingyr crossbred cows [Parâmetros fisiológicos, características morfológicas do pelame e gradientes térmicos de vacas cruzadas holandês-gir]. Bioscience Journal. 2016;32(2):471–477.

Dalcin VC, Fischer V, Daltro DS, Alfonzo EPM, Stumpf MT, Kolling GJ, et al. Physiological parameters for thermal stress in dairy cattle. Revista Brasileira de Zootecnia. 2016;45(8):458–465.

Davison TM, Jonsson NN, Mayer DG, Gaughan JB, Ehrlich WK, McGowan MR. Comparison of the impact of six heat-load management strategies on thermal responses and milk production of feed-pad and pasture fed dairy cows in a subtropical environment. International Journal of Biometeorology. 2016;60(12):1961–1968.

de Palo P, Tateo A, Zezza F, Corrente M, Centoducati P. Influence of Free-Stall Flooring on Comfort and Hygiene of Dairy Cows During Warm Climatic Conditions. Journal of Dairy Science. 2006;89(12):4583–4595.

de Vasconcelos AM, Dias M, Nascimento VA, Façanha DAE. Performance evaluation and adaptability of lactating dairy cows fed soybean and its by-products. Acta Scientiarum - Animal Sciences. 2014;36(4):413–418.

Dikmen S, Alava E, Pontes E., Fear JM, Dikmen BY, Olson TA, et al. Differences in thermoregulatory ability between slick-haired and wild-type lactating Holstein cows in response to acute heat stress. Journal of Dairy Science. 2008;91(9):3395–3402.

Dikmen S, Hansen PJ. Is the temperature-humidity index the best indicator of heat stress in lactating dairy cows in a subtropical environment? Journal of Dairy Science. 2009;92(1):109–116.

Dikmen S, Wang X-Z, Ortega MS, Cole JB, Null DJ, Hansen PJ. Single nucleotide polymorphisms associated with thermoregulation in lactating dairy cows exposed to heat stress. Journal of Animal Breeding and Genetics. 2015;132(6):409–419.

do Amaral BC, Connor EE, Tao S, Hayen J, Bubolz J, Dahl GE. Heat-stress abatement during the dry period: Does cooling improve transition into lactation? Journal of Dairy Science. 2009;92(12):5988–5999.

do Amaral BC, Connor EE, Tao S, Hayen MJ, Bubolz JW, Dahl GE. Heat stress abatement during the dry period influences metabolic gene expression and improves immune status in the transition period of dairy cows. Journal of Dairy Science. 2011;94(1):86–96.

Du Preez JH. Parameters for the determination and evaluation of heat stress in dairy cattle in South Africa. Onderstepoort Journal of Veterinary Research. 2000;67(4):263–271.

Endres MI, Barberg AE. Behavior of Dairy Cows in an Alternative Bedded-Pack Housing System. Journal of Dairy Science. 2007;90(9):4192–4200.

Eslamizad M, Lamp O, Derno M, Kuhla B. The control of short-term feed intake by metabolic oxidation in late-pregnant and early lactating dairy cows exposed to high ambient temperatures. Physiology & Behavior. 2015;145:64–70.

Espinoza JL, Sánchez J, Gracia JA, Sánchez JR, Ortega R, Palacios A. Thermoregulation differs in Chinampo (Bos taurus) and locally born dairy cattle. Turkish Journal of Veterinary and Animal Sciences. 2009;33(3):175–180.

Falk AC, Weary DM, Winckler C, von Keyserlingk MAG. Preference for pasture versus freestall housing by dairy cattle when stall availability indoors is reduced. Journal of Dairy Science. 2012;95(11):6409–6415.

Flamenbaum I, Wolfenson D, Kunz PL, Maman M, Berman A. Interactions Between Body Condition at Calving and Cooling of Dairy Cows During Lactation in Summer. Journal of Dairy Science. octubre de 1995;78(10):2221–2229.

Gallardo MR, Valtorta SE, Leva PE, Gaggiotti MC, Conti GA, Gregoret RF. Diet and cooling interactions on physiological responses of grazing dairy cows, milk production and composition. International Journal of Biometeorology. 2005;50(2):90–95.

Gebremedhin KG, Lee CN, Hillman PE, Collier RJ. Physiological responses of dairy cows during extended solar exposure. In: American Society of Agricultural and Biological Engineers Annual International Meeting 2010, ASABE 2010. 2010. p. 5028–5042.

Gebremedhin KG, Lee CN, Larson JE, Davis J. Alternative cooling of dairy cows by udder wetting. In: American Society of Agricultural and Biological Engineers Annual International Meeting 2012, ASABE 2012. 2012. p. 2073–2092.

Havlin JM, Robinson PH. Intake, milk production and heat stress of dairy cows fed a citrus extract during summer heat. Animal Feed Science and Technology. 2015;208:23–32.

Her E, Wolfenson D, Flamenbaum I, Folman Y, Kaim M, Berman A. Thermal, Productive, and Reproductive Responses of High Yielding Cows Exposed to Short-Term Cooling in Summer. Journal of Dairy Science. 1988;71(4):1085–1092.

Hernández-Rivera JA, Álvarez-Valenzuela FD, Correa-Calderón A, Macías-Cruz U, Fadel JG, Robinson PH, et al. Effect of short-term cooling on physiological and productive responses of primiparous Holstein cows exposed to elevated ambient temperatures. Acta Agriculturae Scandinavica A: Animal Sciences. 2011;61(1).

Honig H, Miron J, Lehrer H, Jackoby S, Zachut M, Zinou A, et al. Performance and welfare of high-yielding dairy cows subjected to 5 or 8 cooling sessions daily under hot and humid climate. Journal of Dairy Science. 2012;95(7):3736–3742.

Igono MO, Johnson HD, Steevens BJ, Krause GF, Shanklin MD. Physiological, Productive, and Economic Benefits of Shade, Spray, and Fan System Versus Shade for Holstein Cows During Summer Heat. Journal of Dairy Science. 1987;70(5):1069–1079.

Jara IE, Keim JP, Arias RA. Behaviour, tympanic temperature and performance of dairy cows during summer season in southern Chile. Archivos de Medicina Veterinaria. 2016;48(1):113–118.

Jonsson NN, McGowan MR, McGuigan K, Davison TM, Hussain AM, Kafi M, et al. Relationships among calving season, heat load, energy balance and postpartum ovulation of dairy cows in a subtropical environment. Animal Reproduction Science. 1997;47(4):315–326.

Kabuga JD, Sarpong K. Influence of weather conditions on milk production and rectal temperature of Holsteins fed two levels of concentrate. International Journal of Biometeorology. 1991;34(4):226–230.

Kanjanapruthipong J, Junlapho W, Karnjanasirm K. Feeding and lying behavior of heat-stressed early lactation cows fed low fiber diets containing roughage and nonforage fiber sources. Journal of Dairy Science. 2015;98(2):1110–1118.

Karimi MT, Ghorbani GR, Kargar S, Drackley JK. Late-gestation heat stress abatement on performance and behavior of Holstein dairy cows. Journal of Dairy Science. 2015;98(10):6865–6875.

Kendall PE, Verkerk GA, Webster JR, Tucker CB. Sprinklers and Shade Cool Cows and Reduce Insect-Avoidance Behavior in Pasture-Based Dairy Systems. Journal of Dairy Science. 2007;90(8):3671–3680.

Ketelaar-de Lauwere CC, Ipema AH, van Ouwerkerk ENJ, Hendriks MMW., Metz JHM, Noordhuizen JPT., et al. Voluntary automatic milking in combination with grazing of dairy cows: Milking frequency and effects on behaviour. Applied Animal Behaviour Science. 1999;64(2):91–109.

Khelil-Arfa H, Faverdin P, Boudon A. Effect of ambient temperature and sodium bicarbonate supplementation on water and electrolyte balances in dry and lactating Holstein cows. Journal of Dairy Science. 2014;97(4):2305–2318.

Khongdee S, Chaiyabutr N, Hinch G, Markvichitr K, Vajrabukka C. Effects of evaporative cooling on reproductive performance and milk production of dairy cows in hot wet conditions. International Journal of Biometeorology. 2006;50(5):253–257.

Khongdee S, Sripoon S, Chousawai S, Hinch G, Chaiyabutr N, Markvichitr K, et al. The effect of modified roofing on the milk yield and reproductive performance of heat-stressed dairy cows under hot-humid conditions. Animal Science Journal. 2010;81(5):606–611.

Kim KH, Kim DH, Oh YK, Lee SS, Lee HJ, Kim DW, et al. Productivity and energy partition of late lactation dairy cows during heat exposure. Animal Science Journal. 2010;81(1):58–62.

Koubková M, Knížková I, Kunc P, Härtlová H, Flusser J, Doležal O. Influence of high environmental temperatures and evaporative cooling on some physiological, hematological and biochemical parameters in high-yielding dairy cows. Czech Journal of Animal Science. 2002;47(8):309–318.

Laporta J, Fabris TF, Skibiel AL, Powell JL, Hayen MJ, Horvath K, et al. In utero exposure to heat stress during late gestation has prolonged effects on the activity patterns and growth of dairy calves. Journal of Dairy Science. 2017;100, 1-9.

Lee CNM, Hillman PE. Thermal responses of Holstein dairy cows on pastures with high solar loads and high winds. In: American Society of Agricultural and Biological Engineers - 6th International Dairy Housing Conference 2007. 2007. p. 253–259.

Legrand AL, von Keyserlingk MAG, Weary DM. Preference and usage of pasture versus free-stall housing by lactating dairy cattle. Journal of Dairy Science. 2009;92(8):3651–3658..

Legrand A, Schütz KE, Tucker CB. Using water to cool cattle: Behavioral and physiological changes associated with voluntary use of cow showers. Journal of Dairy Science. 2011;94(7):3376–3386.

Leyva-Corona JC, Thomas MG, Rincón G, Medrano JF, Correa-Calderón A, Avendaño-Reyes L, et al. Cooling at the summer onset to mitigate the heat stress in Holstein cows from the northwest Mexico. Revista Mexicana De Ciencias Pecuarias. 2016;7(4):415–429.

Li H, Shi Z, Wang C, Ding T, Yan D, Fan L, et al. Evaluating effectiveness of spraying systems to reduce heat stress on dairy cows. In: ASABE - 9th International Livestock Environment Symposium 2012, ILES 2012. 2012. p. 94–99.

Lima IA, de Azevedo M, Borges CRA, Ferreira MA, Guim A, de Almeida GLP. Thermoregulation of Girolando cows during summertime, in Pernambuco State, Brazil. Acta Scientiarum - Animal Sciences. 2013;35(2):193–199.

Lin JC, Moss BR, Koon JL, Flood CA, Rowe S, Martin JR, et al. Effect of Sprinkling Over the Feed Area and Misting Free Stalls on Milk Production1,2,3. The Professional Animal Scientist. 1998;14(2):102–107.

Lohölter M, Meyer U, Rauls C, Rehage J, Dänicke S. Effects of niacin supplementation and dietary concentrate proportion on body temperature, ruminal pH and milk performance of primiparous dairy cows. Archives of Animal Nutrition. 2013;67(3):202–218.

Lough DS, Beede DL, Wilcox CJ. Effects of Feed Intake and Thermal Stress on Mammary Blood Flow and Other Physiological Measurements in Lactating Dairy Cows1. Journal of Dairy Science. 1990;73(2):325–332.

Magdub A, Johnson HD, Belyea RL. Effect of Environmental Heat and Dietary Fiber on Thyroid Physiology of Lactating Cows1. Journal of Dairy Science. 1982;65(12):2323–2331.

Mallonée PG, Beede DK, Collier RJ, Wilcox CJ. Production and Physiological Responses of Dairy Cows to Varying Dietary Potassium During Heat Stress1. Journal of Dairy Science. 1985;68(6):1479–1487.

Marcillac-Embertson NM, Robinson PH, Fadel JG, Mitloehner FM. Effects of shade and sprinklers on performance, behavior, physiology, and the environment of heifers. Journal of Dairy Science. 2009;92(2):506–517.

Matarazzo SV, Silva IJO, Perissinotto M, Moura DJ, Fernandes SAA. Thermal conditioned in resting area of freestall facilities and its consequences on productive and physiological responses in dairy cows. In: 2005 ASAE Annual International Meeting. 2005.

McDowell RE, Moody EG, Van Soest PJ, Lehmann RP, Ford GL. Effect of Heat Stress on Energy and Water Utilization of Lactating Cows. Journal of Dairy Science. 1969;52(2):188–194.

Miron J, Adin G, Solomon R, Nikbachat M, Zenou A, Shamay A, et al. Heat production and retained energy in lactating cows held under hot summer conditions with evaporative cooling and fed two rations differing in roughage content and in vitro digestibility. Animal. 2008;2(6):843–848.

Moallem U, Altmark G, Lehrer H, Arieli A. Performance of high-yielding dairy cows supplemented with fat or concentrate under hot and humid climates. Journal of Dairy Science. 2010;93(7):3192–3202.

Moody EG, Van Soest PJ, McDowell RE, Ford GL. Effect of High Temperature and Dietary Fat on Performance of Lactating Cows. Journal of Dairy Science. 1967;50(12):1909–1916.

Moore CE, Kay JK, Collier RJ, VanBaale MJ, Baumgard LH. Effect of supplemental conjugated linoleic acids on heat-stressed brown Swiss and Holstein cows. Journal of Dairy Science. 2005;88(5):1732–1740.

Neuwirth JG, Norton JK, Rawlings CA, Thompson FN, Ware GO. Physiologic responses of dairy calves to environmental heat stress. International Journal of Biometeorology. 1979;23(3):243–254.

Ominski KH, Kennedy AD, Wittenberg KM, Nia SAM. Physiological and Production Responses to Feeding Schedule in Lactating Dairy Cows Exposed to Short-Term, Moderate Heat Stress. Journal of Dairy Science. 2002;85(4):730–737.

Ominski KH, Wittenberg KM, Kennedy AD, Moshtaghi-Nia SA. Physiological and production responses when feeding Aspergillus oryzae to dairy cows during short-term, moderate heat stress. Animal Science. 2003;77(3):485–490.

Ortiz XA, Smith JF, Bradford BJ, Harner JP, Oddy A. A comparison of the effects of 2 cattle-cooling systems on dairy cows in a desert environment. Journal of Dairy Science. 2010;93(10):4955–4960.

Ortiz XA, Smith JF, Bradford BJ, Harner JP, Oddy A. Effect of complementation of cattle cooling systems with feedline soakers on lactating dairy cows in a desert environment. Journal of Dairy Science. 2011;94(2):1026–1031.

Ortiz XA, Smith JF, Villar F, Hall L, Allen J, Oddy A, et al. A comparison of 2 evaporative cooling systems on a commercial dairy farm in Saudi Arabia. Journal of Dairy Science. 2015a;98(12):8710–8722.

Ortiz XA, Smith JF, Rojano F, Choi CY, Bruer J, Steele T., et al. Evaluation of conductive cooling of lactating dairy cows under controlled environmental conditions. Journal of Dairy Science. 2015b;98(3):1759–1771.

Palacio S, Bergeron R, Lachance S, Vasseur E. The effects of providing portable shade at pasture on dairy cow behavior and physiology. Journal of Dairy Science. 2015;98(9):6085–6093.

Perano KM, Usack JG, Angenent LT, Gebremedhin KG. Production and physiological responses of heat stressed lactating dairy cattle to conductive cooling. En: Journal of dairy science. 2015; 98 (8): 5252–5261.

Pereyra AVG, May VM, Catracchia CG, Herrero MA, Flores MC, Mazzini M. Influence of water temperature and heat stress on drinking water intake in dairy. Chilean Journal of Agricultural Research. 2010;70(2):328–336.

Sailo L, Gupta ID, Verma A, Das R, Chaudhari MV, Singh S. Polymorphisms in Hsp90ab1 gene and their association with heat tolerance in Sahiwal and Karan Fries cows. Indian Journal of Animal Research. 2016;50(6):856–861.

Salvati GGS, Morais Júnior NN, Melo ACS, Vilela RR, Cardoso FF, Aronovich M, et al. Response of lactating cows to live yeast supplementation during summer. Journal of Dairy Science. 2015;98(6):4062–4073.

Schneider PL, Beede DK, Wilcox CJ, Collier RJ. Influence of Dietary Sodium and Potassium Bicarbonate and Total Potassium on Heat-Stressed Lactating Dairy Cows1. Journal of Dairy Science. 1984;67(11):2546–2553.

Schütz KE, Cox NR, Tucker CB. A field study of the behavioral and physiological effects of varying amounts of shade for lactating cows at pasture. Journal of Dairy Science. 2014;97(6):3599–3605.

Shehab-El-Deen MAMM, Leroy JLMR, Fadel MS, Saleh SYA, Maes D, Van Soom A. Biochemical changes in the follicular fluid of the dominant follicle of high producing dairy cows exposed to heat stress early post-partum. Animal Reproduction Science. 2010a;117(3-4):189–200.

Shehab-El-Deen MAMM, Fadel MS, van Soom A, Saleh SY, Maes D, Leroy JLMR. Circadian rhythm of metabolic changes associated with summer heat stress in high-producing dairy cattle. Tropical Animal Health and Production. 2010b;42(6):1119–1125.

Shwartz G, Rhoads ML, Vanbaale MJ, Rhoads RP, Baumgard LH. Effects of a supplemental yeast culture on heat-stressed lactating Holstein cows. Journal of Dairy Science. 2009;92(3):935–942.

Smith J, Bradford B, Harner J, Potts J, Allen J, Overton M, et al. Short communication: Effect of cross ventilation with or without evaporative pads on core body temperature and resting time of lactating cows. Journal of Dairy Science. febrero de 2016;99(2):1495–1500.

Soriani N, Panella G, Calamari L. Rumination time during the summer season and its relationships with metabolic conditions and milk production. Journal of Dairy Science. 2013;96(8):5082–5094.

Spiers DE, Spain JN, Sampson JD, Rhoads RP. Use of physiological parameters to predict milk yield and feed intake in heat-stressed dairy cows. Journal of Thermal Biology. 2004;29(7–8):759–764.

Stone AE, Jones BW, Becker CA, Bewley JM. Influence of breed, milk yield, and temperature-humidity index on dairy cow lying time, neck activity, reticulorumen temperature, and rumination behavior. Journal of Dairy Science. 2017;100(3):2395–2403.

Su H, Wang Y, Zhang Q, Wang F, Cao Z, Rahman MAU, et al. Responses of energy balance, physiology, and production for transition dairy cows fed with a low-energy prepartum diet during hot season. Tropical Animal Health and Production. 2013;45(7):1495–1503.

Tao S, Bubolz J, do Amaral B, Thompson I, Hayen M, Johnson S, et al. Effect of heat stress during the dry period on mammary gland development. Journal of Dairy Science. 2011;94(12):5976–5986.

Tao S, Thompson IM, Monteiro APA, Hayen MJ, Young LJ, Dahl GE. Effect of cooling heat-stressed dairy cows during the dry period on insulin response. Journal of Dairy Science. septiembre de 2012;95(9):5035–5046.

Tapkı İ, Şahin A. Comparison of the thermoregulatory behaviours of low and high producing dairy cows in a hot environment. Applied Animal Behaviour Science. 2006;99(1–2):1–11.

Thompson IMT, Tao S, Monteiro APA, Jeong KC, Dahl GE. Effect of cooling during the dry period on immune response after Streptococcus uberis intramammary infection challenge of dairy cows. Journal of Dairy Science. 2014;97(12):7426–7436.

Titto C, Negrão J, Titto E, Canaes T, Titto R, Pereira A. Effects of an evaporative cooling system on plasma cortisol, IGF-I, and milk production in dairy cows in a tropical environment. International Journal of Biometeorology. 2013;57(2):299–306.

Tresoldi G, Schütz KE, Tucker CB. Assessing heat load in drylot dairy cattle: Refining on-farm sampling methodology. Journal of Dairy Science. 2016;99(11):8970–8980.

Tripon I, Cziszter L, Bura M, Sossidou E. Effects of seasonal and climate variations on calves’ thermal comfort and behaviour. International Journal of Biometeorology. 2014;58(7):1471–1478.

Tsai YC, Castillo LS, Hardison WA, Payne WJA. Effect of Dietary Fiber Level on Lactating Dairy Cows in the Humid Tropics. Journal of Dairy Science. 1967;50(7):1126–1129.

Tucker CB, Rogers AR, Schütz KE. Effect of solar radiation on dairy cattle behaviour, use of shade and body temperature in a pasture-based system. Applied Animal Behaviour Science. 2008;109(2–4):141–154.

Valtorta SE, Leva PE, Gallardo MR. Evaluation of different shades to improve dairy cattle well-being in Argentina. International Journal of Biometeorology. 1997;41(2):65–67.

Valtorta S, Gallardo M. Evaporative cooling for Holstein dairy cows under grazing conditions. International Journal of Biometeorology. 2004;48(4):213–217.

Vizzotto E, Fischer V, Thaler Neto A, Abreu A, Stumpf M, Werncke D, et al. Access to shade changes behavioral and physiological attributes of dairy cows during the hot season in the subtropics. Animal. 2015;9(9):1559–1566.

Wang J, Bu D, Wang J, Huo X, Guo T, Wei H, et al. Effect of saturated fatty acid supplementation on production and metabolism indices in heat-stressed mid-lactation dairy cows. Journal of Dairy Science. 2010;93(9):4121–4127.

West JW, Mullinix BG, Sandifer TG. Changing Dietary Electrolyte Balance for Dairy Cows in Cool and Hot Environments1. Journal of Dairy Science. 1991;74(5):1662–1674.

West J, Hill G, Fernandez J, Mandebvu P, Mullinix BG. Effects of dietary fiber on intake, milk yield, and digestion by lactating dairy cows during cool or hot, humid weather. Journal of Dairy Science. 1999;82(11):2455–2465.

West JW, Mullinix BG, Bernard JK. Effects of hot, humid weather on milk temperature, dry matter intake, and milk yield of lactating dairy cows. Journal of dairy science. 2003;86(1):232–242.

Wheelock JB, Rhoads RP, VanBaale MJ, Sanders SR, Baumgard LH. Effects of heat stress on energetic metabolism in lactating Holstein cows1. Journal of Dairy Science. 2010;93(2):644–655.

Wise ME, Armstrong DV, Huber JT, Hunter R, Wiersma F. Hormonal alterations in the lactating dairy cow in response to thermal stress. Journal of dairy science. 1988;71(9):2480–2485.

Wolfenson D, Flamenbaum I, Berman A. Hyperthermia and Body Energy Store Effects on Estrous Behavior, Conception Rate, and Corpus Luteum Function in Dairy Cows. Journal of Dairy Science. 1988;71(12):3497–3504.

Yan G, Wang C, Li B, Zhang G, Shi Z, Li H, et al. Influence of water temperature and spraying interval on cooling effect of sprinkler system in dairy barns. Applied Engineering in Agriculture. 2014;30(4):611–617.

Younas M, Fuquay JW, Smith AE, Moore AB. Estrous and Endocrine Responses of Lactating Holsteins to Forced Ventilation During Summer1. Journal of Dairy Science. 1993;76(2):430–436.

Zähner M, Schrader L, Hauser R, Keck M, Langhans W, Wechsler B. The influence of climatic conditions on physiological and behavioural parameters in dairy cows kept in open stables. Animal Science. 2004;78(1):139–147.

Zimbelman RB, Baumgard LH, Collier RJ. Effects of encapsulated niacin on evaporative heat loss and body temperature in moderately heat-stressed lactating Holstein cows. Journal of Dairy Science. 2010;93(6):2387–2394.
